# Supplementary material for: Integration of Metabolomes and Transcriptomes Provides Insights into Morphogenesis and Maturation in Morchella sextelata
Source: J Fungi (Basel). 2023 Nov 27;9(12):1143. doi: 10.3390/jof9121143 (PMC10744280; doi:10.3390/jof9121143)
Supplement: Supplementary file 1 [file jof-09-01143-s001.zip › Supplementary figures.pdf]

## Supplemental figures

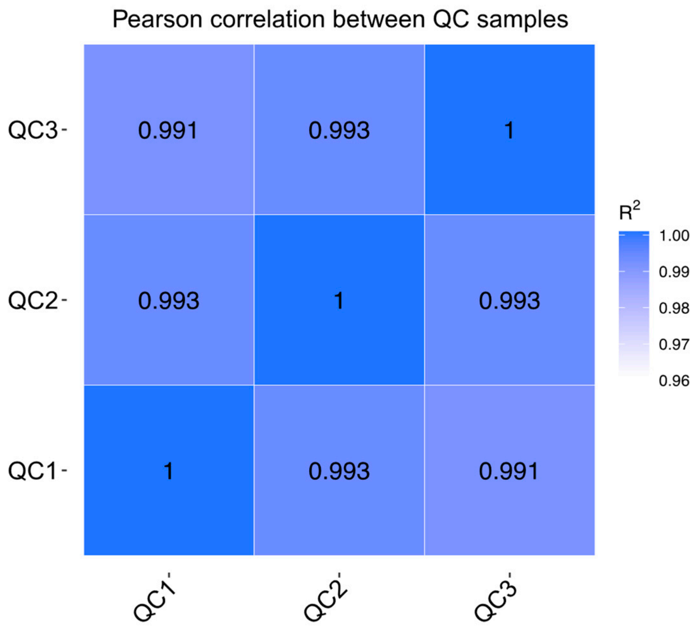


**Figure S1. Pearson correlation analysis between QC samples.**

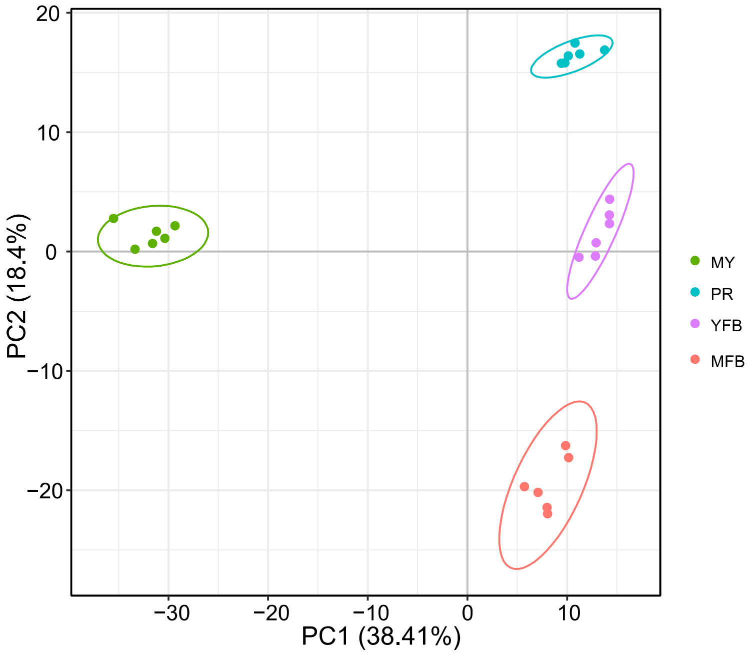


**Figure S2. PLS-DA analysis of metabolites composition at different stages.**

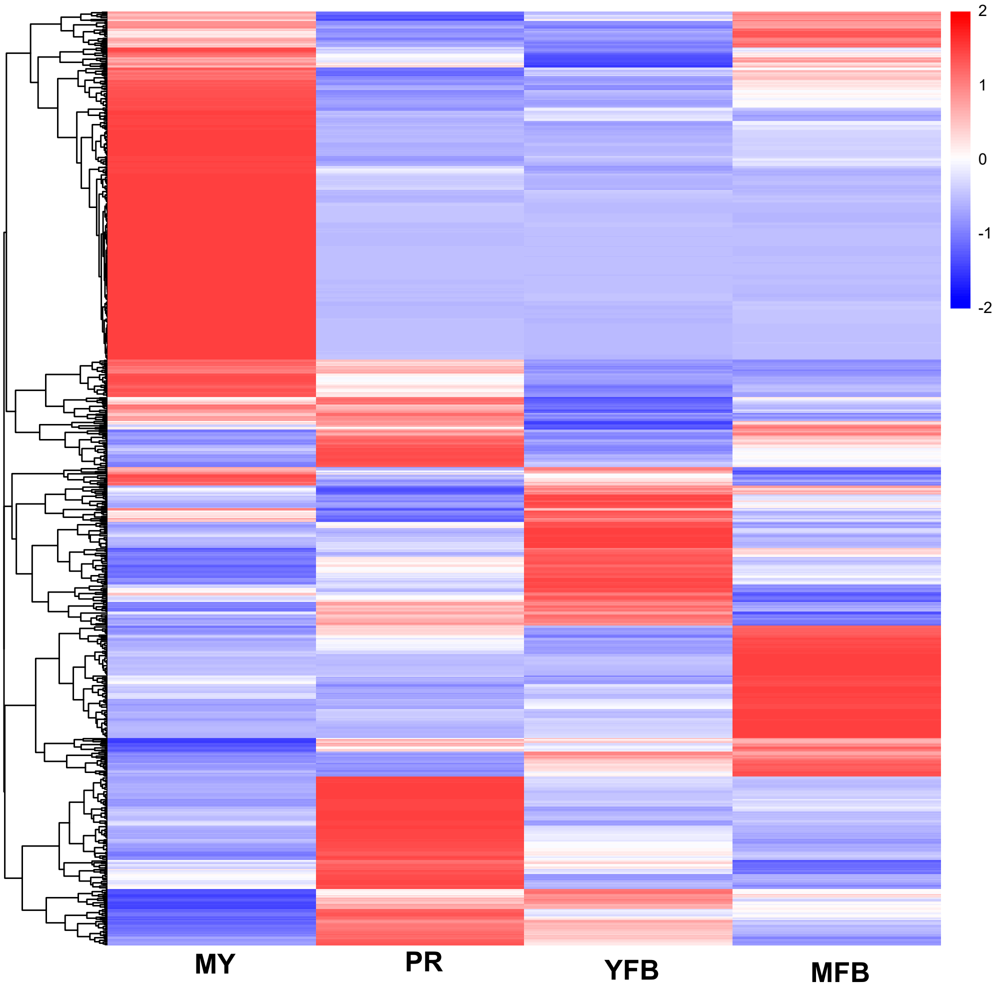


**Figure S3. Heatmap showing the DEMs at different stages.**

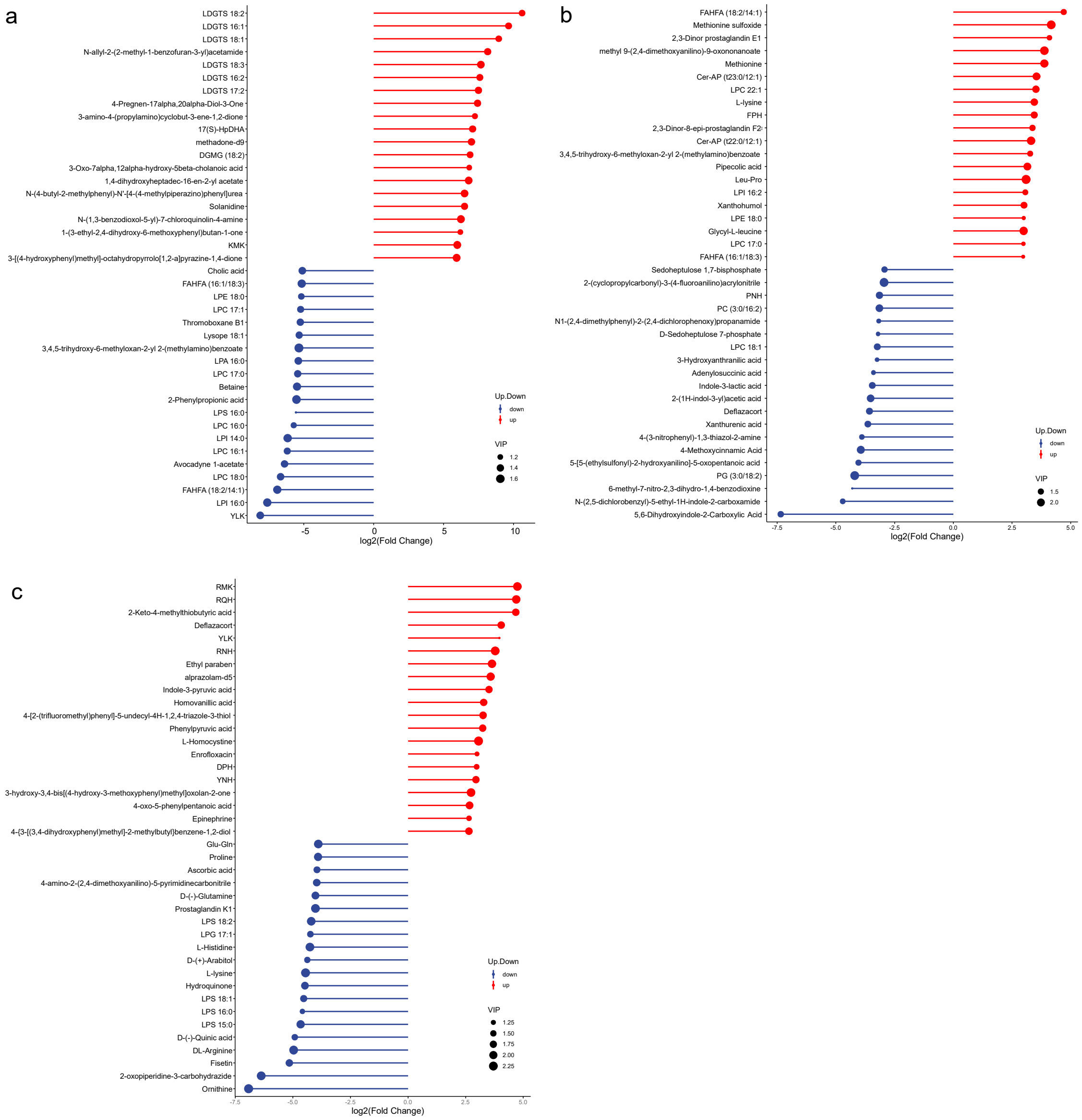


**Figure S4. Top 20 DEMs in three comparative groups. (a) MY vs. PR. (b) PR vs. YFB. (c) YFB vs. MFB.**

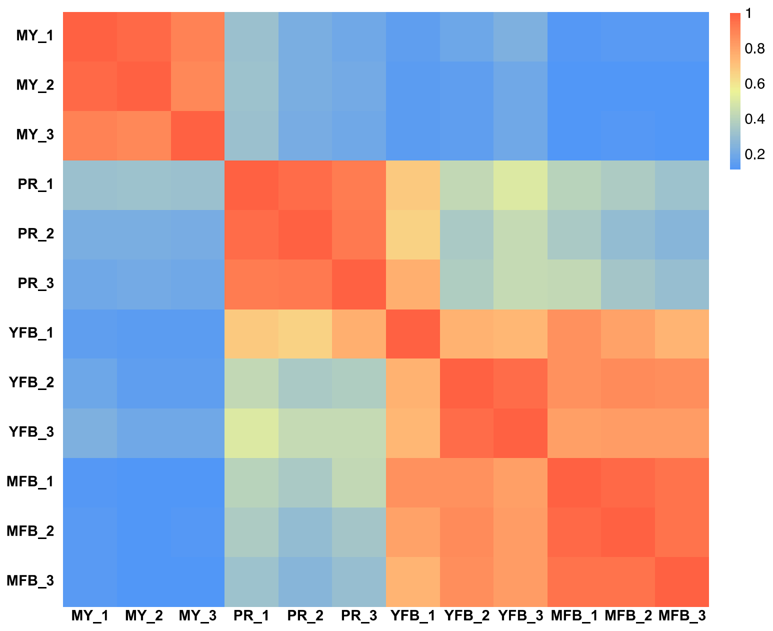


**Figure S5. Pearson correlation analysis between all transcriptome samples.**

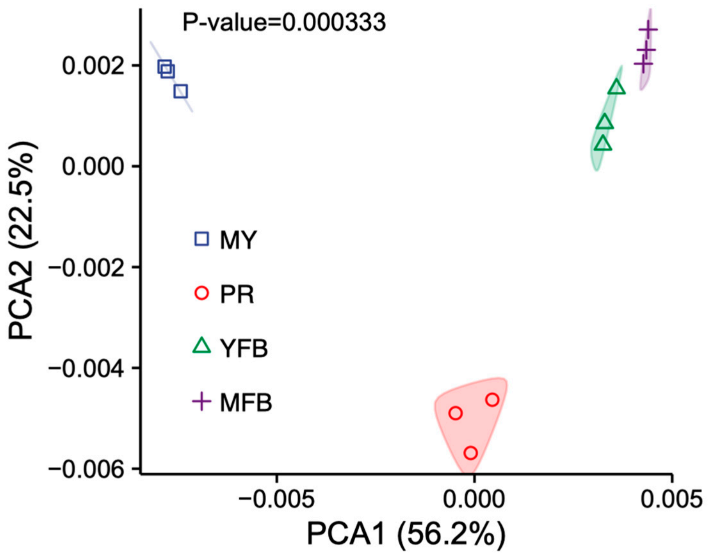


**Figure S6. PCA analysis of gene expression profiles at different stages.**

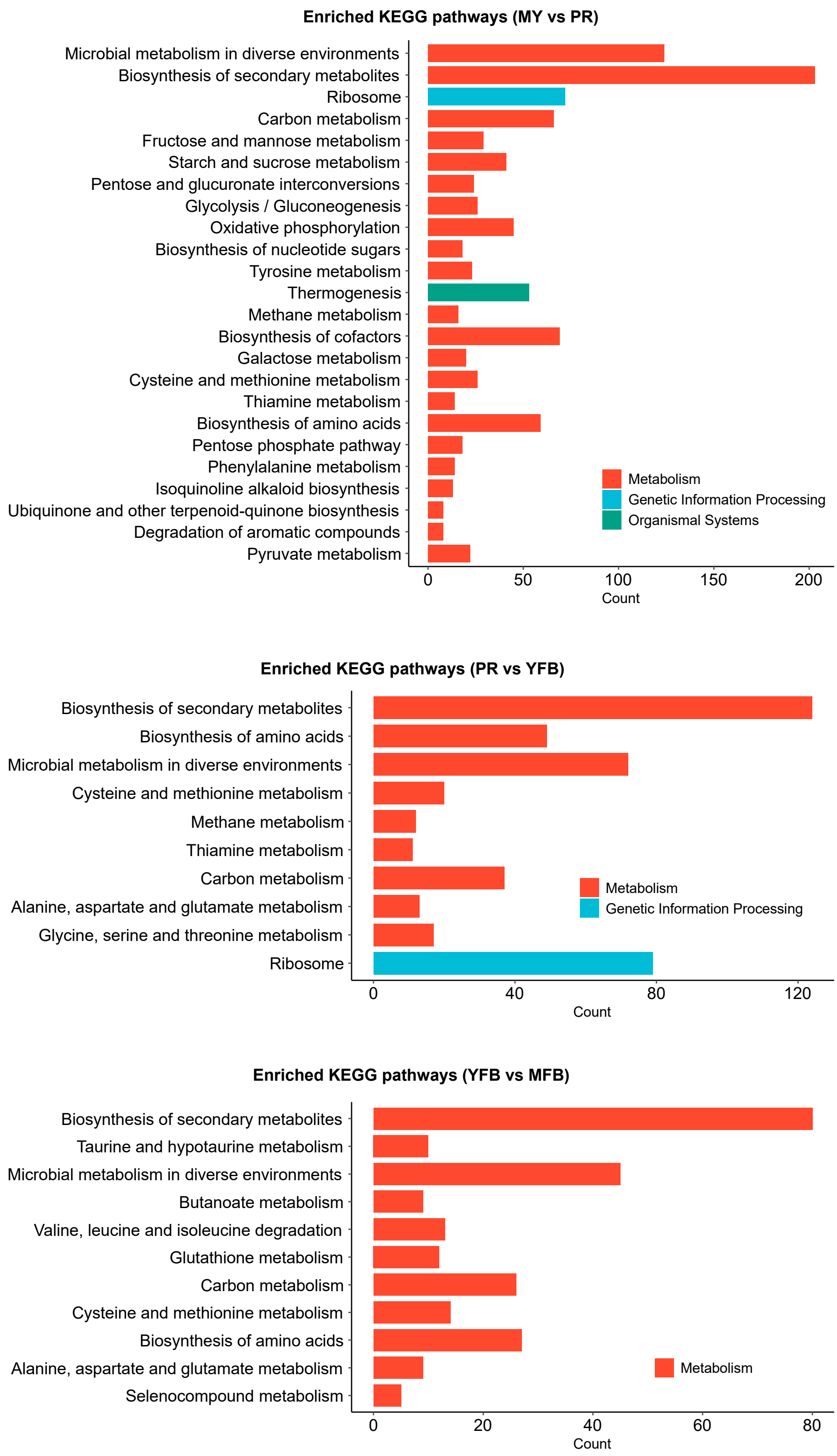


**Figure S7. KEGG enrichment analysis of DEGs in MY vs. PR, PR vs. YFB and YFB vs. MFB.**

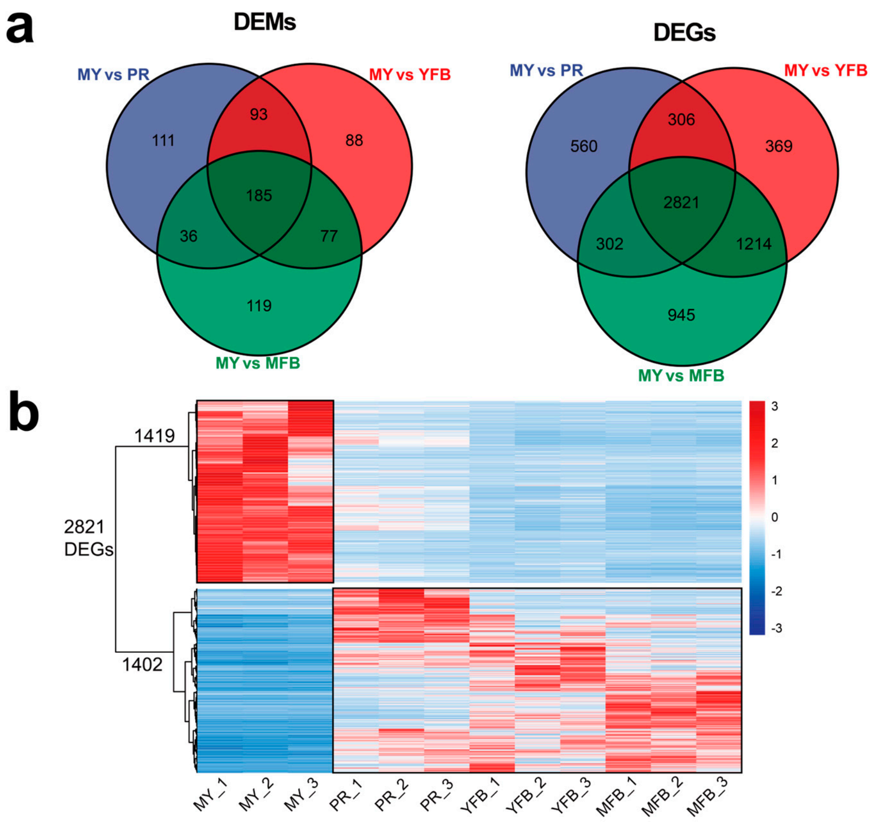


**Figure S8. (a) Venn diagram of DEMs and DEGs in MY vs. PR, MY vs. YFB and MY vs. MFB. (b) Expression heatmap of 2,821 shared DEGs in MY vs. PR, MY vs. YFB and MY vs. MFB.**

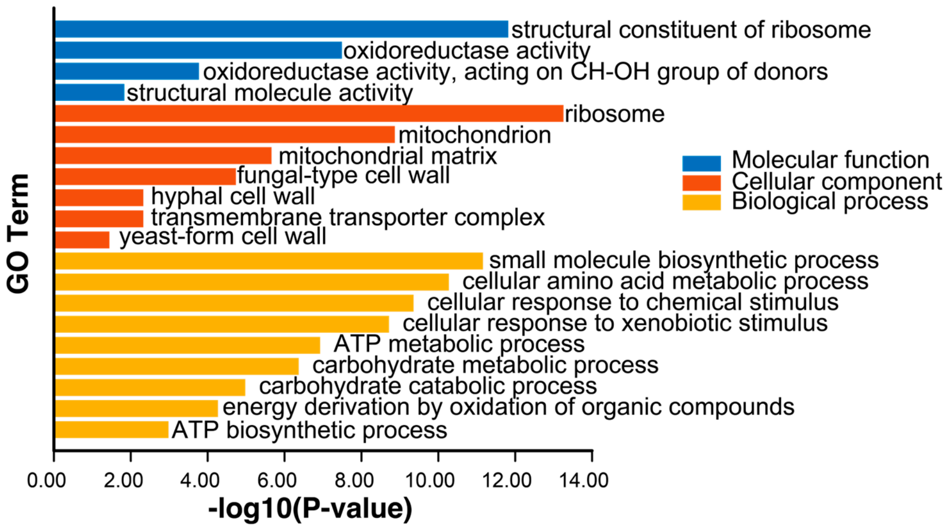


**Figure S9. Enriched GO terms of 1,402 upregulated DEGs in PR, YFB and MFB stages.**

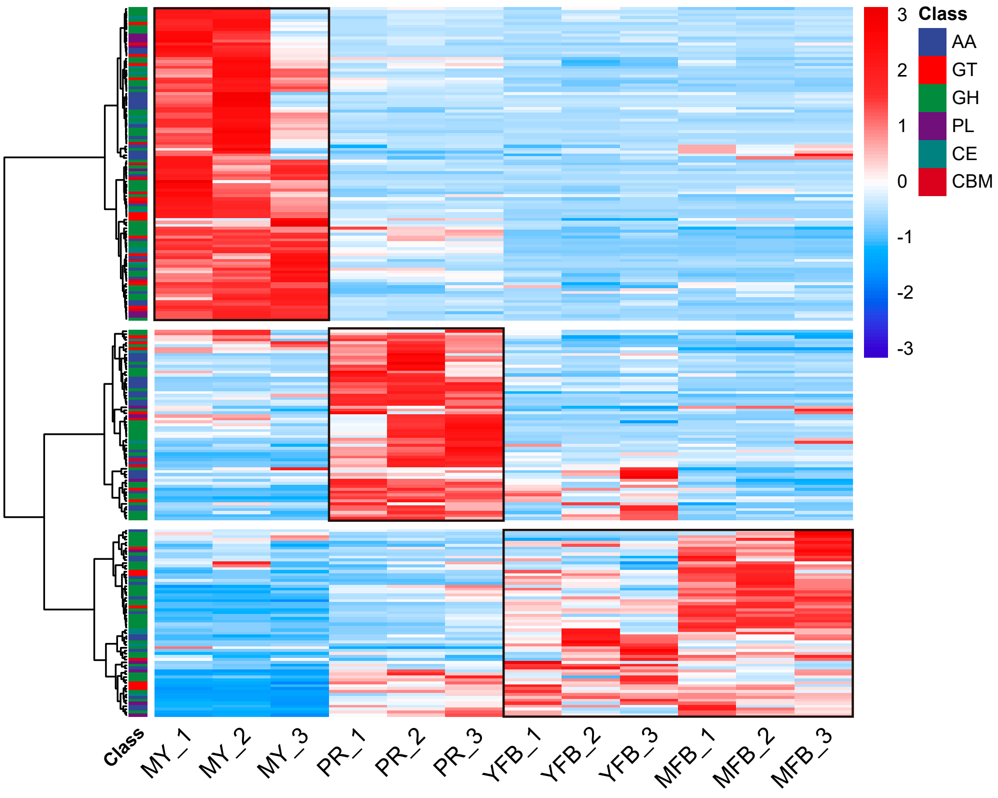


**Figure S10. Heatmap showing differentially expressed CAZymes at different developmental stages.**

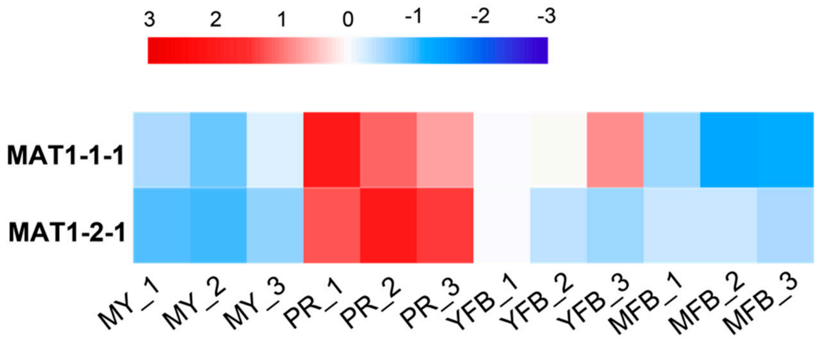


**Figure S11. Heatmap showing differentially expressed mating-type genes at different developmental stages.**
